# ﻿Isolation and characterization of edible mushroom-forming fungi from Swedish nature

**DOI:** 10.3897/imafungus.16.142215

**Published:** 2025-02-17

**Authors:** Mario Walthert, Markus Hiltunen Thorén, Hanna Johannesson

**Affiliations:** 1 Department of Ecology, Environment and Plant Sciences, Stockholm University, Svante, Arrhenius väg 20, 104 05 Stockholm, Sweden Stockholm University Stockholm Sweden; 2 The Royal Swedish Academy of Sciences, Lilla Frescativägen 4A, 114 18 Stockholm, Sweden The Royal Swedish Academy of Sciences Stockholm Sweden

**Keywords:** Fungal biotechnology, mushroom cultivation, mycelial growth rate, optimal growth temperature, primordia initiation, Swedish strains

## Abstract

Fungi are a highly diverse group of organisms, of which only a small subset has been taken into cultivation for application in biotechnology and food industry. Accordingly, outside of a few model species, there is a lack of knowledge about the isolation and cultivation of fungi. In this study, we isolated 17 wild strains of 14 different species of edible, mushroom-forming fungi growing in Swedish nature. We documented their growth rates under different temperatures, investigated their fruiting characteristics, and compared the results to data obtained from common laboratory strains. Our results show that the strains from commercially cultivated species have a higher mycelial growth rate and tend to grow faster at higher temperatures than strains from less frequently cultivated species. The fruiting experiments led to successful fruiting of four newly collected wild strains, belonging to the species *Hericiumcoralloides*, *Pleurotuspulmonarius*, and *Schizophyllumcommune*. Although some strains fruited on potato dextrose agar (PDA), more specific substrates such as straw or birch pellets indicated more potential for mushroom production. All newly isolated strains of this study have been deposited at the Westerdijk Fungal Biodiversity Institute (CBS) collection and are thereby made available for further studies and/or use in application in the food industry or biotechnology. Two species isolated in this study are entirely novel to widely used culture collections, and for nine species no Swedish strain has been deposited previously. The description of the mycelial growth and fruiting of the isolated strains in this study is a first step on their way to further use.

## ﻿Introduction

One of the most popular uses of fungi is mushrooms as a food product. Both wild and cultivated fungi provide resources of mushrooms that are rich in both flavors and health benefits (Barros et al. 2008). They constitute a low-calorie source of fiber, protein, vitamins and minerals (Dimopoulou et al. 2022). Wild edible fungi are collected for food each year in several million tons (Royse et al. 2017), yet the global mushroom production is largely dominated by cultivated edible mushrooms (Niego et al. 2023; see data on trade flows at the https://comtradeplus.un.org). Artificial mushroom cultivation is done on substrates that mimic the naturally occurring habitats of fungi. Sourcing these cultivation substrates, for example wood particles or seed hulls, from waste streams of forestry, agriculture, and other industrial branches can contribute to a more circular economy (Grimm and Wösten 2018; Meyer et al. 2020; Kržišnik and Gonçalves 2023). The substrate determines the success of mycelial growth and fruiting body development together with other cultivation conditions (Stamets 2000; Miles and Chang 2004; Sánchez 2010; Sakamoto 2018). Saprotrophic species are naturally more amenable to cultivation than mycorrhizal species, since the former are not dependent on a symbiotic partner to complete their life cycle (Thawthong et al. 2014; Sakamoto 2018; Guerin-Laguette 2021).

The species cultivated in the food industry today represent only the tip of the iceberg regarding diversity of edible mushroom-forming fungi. Only a few species are responsible for more than 70% of the global mushroom production: *Agaricusbisporus* (Button mushroom), *Lentinulaedodes* (Shiitake), and *Pleurotus* spp. (Oyster mushrooms) (Boa 2004; Chakravarty 2011). However, the increase in global consumption and emerging species on the market such as *Hericiumerinaceus* (Lion’s Mane) show that consumers are open for more species (Miles and Chang 2004; Sánchez 2010; Li et al. 2021). The fungal kingdom bears many more cultivatable species, potentially even suitable for commercial scale cultivation (Boa 2004; Hyde et al. 2019; Li et al. 2021). The availability of a diverse set of strains from different parts of the world is particularly interesting, as the demand for local products, e.g. in restaurants, is rising (Thawthong et al. 2014; Hellwig 2023).

Before new strains are cultivated, they need to be isolated from nature into pure culture, and their optimal growth conditions need to be determined. Gathering information on growth preferences is even more important considering the limited scientific knowledge for the majority of species and the complexity of mushroom cultivation. Characterization of growth conditions for new fungal strains is the first step towards their application in the food industry and biotechnology. The aim of this study was to isolate new strains from a diverse set of edible saprotrophic, mushroom-forming fungal species in Swedish forests and meadows, and to investigate their mycelial growth and possible fruiting body development in various cultivation conditions.

## ﻿Material and methods

### ﻿Sampling

Sampling took place in the Stockholm-Uppsala area of central Sweden during the interval of May 2023 to April 2024 (Table 1). The only exception was *Marasmiusoreades* MW66 which originates from a mushroom which was collected, isolated as monokaryotic single basidiospore isolates in 2021, and subsequently stored at 4 °C. The sampling effort was designed to isolate strains from as many different fungal species as possible. Conservation was considered when mushrooms were sampled. We limited the impact on the organism and the environment by never sampling more than three mushrooms per site, and no threatened species was sampled (Naturvårdsverket 2016; IUCN 2023).

### ﻿Isolation of pure culture

For the sampled wild strains, we applied either of two methods to obtain a pure mycelium culture (Suppl. material 1: fig. S1; Table 1). Culturing attempts were in all of these cases done within 48 hours of sampling. In the “tissue isolation method” (Suppl. material 1: fig. S1), the mushroom stipe was first surface sterilized by submergence for 10 seconds in 70% ethanol. Afterwards, inner stipe tissue was placed onto fresh potato dextrose agar (PDA, NutriSelect® Basic, Merck KGaA, Darmstadt, Germany; 4 g L^-1^ potato extract, 20 g L^-1^ dextrose, 15 g L^-1^ agar) plates supplemented with 10 ml L^-1^ Penicillin-Streptomycin (BioReagent, Sigma-Aldrich, Burlington, USA; 10’000 U ml^-1^ penicillin, 10 mg ml^-1^ streptomycin) and incubated at 20 °C without light in a *MLR-351H* incubator (Sanyo, Moriguchi, Japan). When hyphae emerged from the tissue, they were transferred onto a new PDA plate to evade contaminants. After several rounds of subculturing, we obtained a pure monohyphal culture by cutting off and transferring a single hyphal tip from the subculture mycelium growth front to a fresh plate. For the second method, the “spore print isolation method” (Suppl. material 1: fig. S1), mushroom caps were placed with the spore-bearing structure facing downwards onto a sterile petri dish (92 mm, polystyrene, Sarstedt, Nümbrecht, Germany). After 12–48 hours, the collected basidiospores were suspended in sterilized water, and 100 µl of the suspension were streaked onto a PDA plate with 10 ml L^-1^ Penicillin-Streptomycin. The germinated spores were identified by eye under a stereo microscope and isolated by picking with a sterile needle and transferring to a fresh PDA plate. To create a pure dikaryotic culture, mycelium from two single-basidiospore monokaryons was transferred onto the same PDA plate. Their compatibility and hyphal fusion to a dikaryotic mycelium was confirmed by the observation of clamp connections, which is a common method to distinguish a dikaryon from a monokaryon in basidiomycetes (Miles and Chang 2004).

For *M.oreades*, we continued with the single-spore isolates as for the other strains with the spore print isolation method. The only ascomycete strains in this project, *Morchellaimportuna* Zebra-I and Zebra-II, were isolated from dry morel fruiting body tissue collected in May 2023. For both *M.importuna* strains, we revitalized the dry tissue by dipping it for 10 seconds in sterilized water, then surface sterilized it with 70% ethanol, and washed it with sterilized water three times for 10 seconds. The sterilized morel tissue was then further treated as other wild mushroom tissue in the above-mentioned tissue isolation method.

**Table 1.** List of wild strains isolated in this study.

Strain name	Sampling site^a^	Coordinates	Sampling date	Sampled by	Preliminary species determination^b^	Isolation method	Matched GenBank Accession	Seq. length^c^ (bp)	Species^d^	Identity (%)	CBS numbere	GenBank Accession
**Basidiomycetes**												
*Agaricusarvensis* MW41	Stocksund, Stockholm	59°22'58.7"N, 18°02'56.1"E	29.09.2023	Mario Walthert	*Agaricus* sp.	Tissue	MH861005.1	665	*Agaricusarvensis*	100.00	152132	PP944384
*Armillariaborealis* MW40	Hågadalen, Uppsala	59°49'45.1"N, 17°35'24.3"E	12.09.2023	Mario Walthert	*Armillaria* sp.	Tissue	MW418559.1	810	*Armillariaborealis*	99.50	152131	PP944356
*Armillariaborealis* MW82	Hågadalen, Uppsala	59°49'38.1"N, 17°35'19.2"E	12.09.2023	Mario Walthert	*Armillaria* sp.	Tissue	MW418559.1	788	*Armillariaborealis*	100.00	152138	PP944586
*Coprinuscomatus* MW21	Djurgården, Stockholm	59°21'52.1"N, 18°03'39.9"E	24.10.2023	Mario Walthert	*Coprinuscomatus*	Tissue	MK169229.1	586	*Coprinuscomatus*	99.83	152129	PP944328
*Hericiumcoralloides* MW80	Hågadalen, Uppsala	59°49'41.3"N, 17°35'14.3"E	12.09.2023	Mario Walthert	*Hericiumcoralloides*	Tissue	OM033735.1	470	*Hericiumcoralloides*	99.57	152137	PP944580
*Hypholomalateritium* MW49	Djurgården, Stockholm	59°21'58.5"N, 18°03'58.7"E	17.10.2023	Mario Walthert	*Hypholomalateritium*	Tissue	KX449453.1	620	*Hypholomalateritium*	99.68	152134	PP944491
*Lycoperdonperlatum* MW73	Hågadalen, Uppsala	59°49'45.4"N, 17°35'22.5"E	12.09.2023	Mario Walthert	*Lycoperdonperlatum*	Tissue	OM044595.1	663	*Lycoperdonperlatum*	99.70	152203	PP944581
*Macrolepiotaprocera* MW90	Flottsund, Uppsala	59°46'19.8"N, 17°38'26.6"E	14.10.2023	Hanna Johannesson	*Macrolepiotaprocera*	Tissue	MK169238.1	643	*Macrolepiotaprocera*	99.53	152139	PP944587
*Marasmiusoreades* MW66	Ultuna, Uppsala	59°48'45.9"N, 17°39'40.0"E	01.09.2022	Markus Hiltunen	*Marasmiusoreades*	Spore print	KY366495.1	645	*Marasmiusoreades*	99.84	152136	PP944579
*Paralepistaflaccida* MW6	Stocksund, Stockholm	59°23'29.5"N, 18°03'46.6"E	17.10.2023	Mario Walthert	*Paralepistaflaccida*	Tissue	MK169239.1	614	*Paralepistaflaccida*	99.51	152130	PP944300
*Pleurotusostreatus* MW97	Djursholm, Stockholm	59°23'13.2"N, 18°05'10.8"E	01.04.2024	Mario Walthert	*Pleurotus* sp.	Tissue	MT644908.1	565	*Pleurotusostreatus*	99.47	152140	PP944612
*Pleurotuspulmonarius* MW44	Djurgården, Stockholm	59°22'02.7"N, 18°03'38.3"E	03.10.2023	Mario Walthert	*Pleurotus* sp.	Spore print	KY962457.1	592	*Pleurotuspulmonarius*	99.66	152133	PP944406
*Schizophyllumcommune* KK98	Bergshamra, Stockholm	59°22'30.8"N, 18°02'13.2"E	23.03.2024	Katerina Krajinova	*Schizophyllumcommune*	Tissue	MN341837.1	552	*Schizophyllumcommune*	99.82	152141	PP944615
*Schizophyllumcommune* MW99	Bergshamra, Stockholm	59°22'31.0"N, 18°02'11.9"E	23.03.2024	Mario Walthert	*Schizophyllumcommune*	Tissue	MN341837.1	561	*Schizophyllumcommune*	99.82	152142	PP944613
*Strophariaaeruginosa* MW56	Djurgården, Stockholm	59°21'52.7"N, 18°05'35.7"E	17.10.2023	Mario Walthert	*Strophariaaeruginosa*	Tissue	KX449459.1	610	*Strophariaaeruginosa*	99.67	152135	PP944574
***Ascomycetes***												
*Morchellaimportuna* Zebra-I	Mörkhultet, Jönköping	57°26'28.6"N, 14°14'43.5"E	01.05.2023	Zebra Ousbäck	*Morchella* sp.	Tissue	MH982774.1	653	*Morchellaimportuna*	99.23	152143	PP945886
*Morchellaimportuna* Zebra-II	Mörkhultet, Jönköping	57°26'28.1"N, 14°14'51.1"E	01.05.2023	Zebra Ousbäck	*Morchella* sp.	Tissue	MH982774.1	651	*Morchellaimportuna*	99.69	152144	PP946098

^a^ All sampling sites are located in central Sweden. ^b^ Based on morphological determination in the field. ^c^ Sequence length of the wild strain ITS sequence used for the GenBank BLAST ^d^ Based on species of GenBank accession with the highest identity to wild strain ITS sequence. ^e^ Number at the Westerdijk Fungal Biodiversity Institute (CBS) culture collection. TBA = to be announced as soon as we have the information.

### ﻿ITS sequencing

To ensure that the cultures were pure, and that the mycelium was from the mushroom origin, we amplified and sequenced the internal transcribed spacer region (ITS); the official barcoding marker for fungi (Schoch et al. 2012; Raja et al. 2017). For this purpose, we first grew the pure culture mycelium on PDA covered with cellophane, and harvested the mycelial tissue by scraping it off the plate with a sterile cell scraper. DNA was extracted from the tissue by using the Quick-DNA Fungal/Bacterial Miniprep kit (Zymo Research Corporation, Irvine, USA) and the extracted DNA was used to amplify the ITS sequence with *ITS1* and *ITS4* primers (White et al. 1990). The PCR reaction contained 1 μl DNA, 4 μl 5× ﻿Phusion HF Buffer, 0.4 μl 10 mM dNTP, 0.5 μl of each primer (20 μM), 0.6 μl DMSO, 0.2 μl ﻿Phusion Polymerase, and milliQ water to a final volume of 20 μl per reaction. The PCR ﻿program consisted of initial denaturation for 1 min at 98 °C; 35 cycles of 15 s at 98 °C, 30 s at 58 °C, and 45 s at 72 °C; and a 7 min extension phase at 72 °C.

PCR products were cleaned using the QIAquick PCR Purification kit (QIAGEN, Venlo, The Netherlands). Cleaned PCR products were sequenced by Eurofins Genomics Germany GmbH, using Sanger technology and the same primers as were used for amplification. The resulting chromatograms were visually inspected, and regions of low quality were removed. A consensus ITS-sequence was generated for each strain using BioEdit (Hall 1999). Sequences similar to the obtained ITS sequences were searched for in the NCBI GenBank NT database using BLASTN (Sayers et al. 2021). The result from the BLAST search was compared with the mushroom morphology-based species preliminary determination in the field, to check whether a mycelium from other than the target species was isolated. Finally, the newly generated ITS sequences were submitted to NCBI together with information on the strain (Table 1).

### ﻿Control strains

To test whether our experimental setup for growth and fruiting body formation was suitable, and for comparative purposes, we included six control strains from five species of basidiomycetes in our study (*Coprinopsiscinerea*, *Flammulinavelutipes*, *Pleurotusostreatus*, *Pleurotuspulmonarius*, and *Schizophyllumcommune*; Suppl. material 2: table S1). These are all established species in mushroom production or research, and thus have documented cultivation protocols (Stamets 2000; Miles and Chang 2004; Kamada et al. 2010; Sánchez 2010; Kües et al. 2016; Grimm and Wösten 2018; Sakamoto 2018; Singh et al. 2021). The control strains were provided by research institutions or companies (Suppl. material 2: table S1).

### ﻿Determination of optimal growth temperatures

We investigated the optimal growth temperature (OGT) by comparing mycelial growth on PDA plates incubated at different temperatures, and identified OGT as a temperature, or a range of temperatures, that resulted in significantly higher growth rates compared to both higher and lower temperatures. First, we tested mycelial growth of each strain on PDA at 20, 25, and 30 °C. Based on the result of this initial screen, we also, when needed, investigated growth at lower or higher temperatures (10, 15, or 35 °C) to identify the growth optimum.

**Figure 1.**
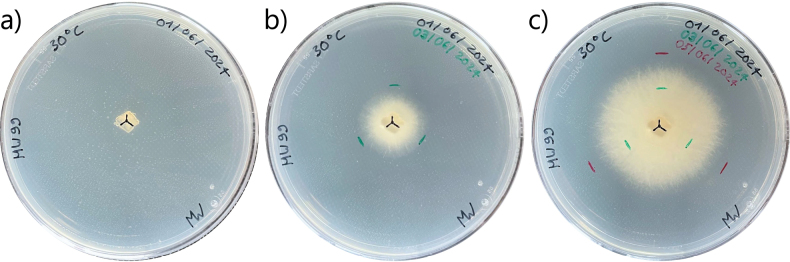
Marking of the mycelial growth front on the plate bottom **a** plate after inoculation, with marking of inoculation center with three previously defined measuring directions (black) **b** second marking (green) showing mycelium front after initial growth **c** third marking (red) showing mycelium front at end of experiment.

For the inoculation, approximately 5 × 5 mm cubes of the pure cultures’ growth front were cut with a lancet-shaped dissection needle and placed in the center of fresh PDA plates. We inoculated three replicate plates per strain for each temperature, i.e. we used three biological replicates for each measurement. We marked the center of the inoculation point and three measurement directions (Fig. 1a), whereafter the plates were sealed with Parafilm®, stacked and placed in poly propylene boxes (40 × 30 × 18 cm, Orthex Sweden AB, Tingsryd, Sweden) together with a glass beaker filled with 300 ml of water to avoid dry conditions, and incubated at the designated temperature.

Mycelial growth was assessed with the radial mycelial growth rate. To minimize the effect of inoculum size and to enter the phase of linear growth, we made a second marking to indicate the starting of the assay when the mycelial growth front on the petri dish had expanded at least three millimeters from the inoculum (Fig. 1b; Brancato and Golding 1953; Taniwaki et al. 2006). A third marking was made after the growth front moved at least another five millimeters outwards (Fig. 1c). The distances between the second and the third marking were measured and averaged over the three directions. We divided this average distance by the number of days to get the radial growth rate for each plate, i.e. each biological replicate.

### ﻿Statistical analysis

The statistical analysis was performed in R 4.1.1 (R Core Team 2021). The growth rate data separated by strain were tested for non-normality with the Shapiro-Wilk test (Shapiro and Wilk 1965). An insignificant Shapiro-Wilk test with the confidence level of 95% indicated that the growth rate was normally distributed, and for these strains, we performed a linear model and analysis of variance (ANOVA) to infer differences in growth between each temperature. The model was then tested for significant between-temperature differences with the Tukey multiple comparison of means test (Tukey 1959). We plotted the results with significance letter grouping using the multcompView package in R (Graves et al. 2023). For strains for which the Shapiro-Wilk test indicated non-normal data distribution, we chose a cluster analysis to infer the OGT. The cluster analysis was done with the kmeans and silhouette function of the cluster package (Maechler et al. 2023). The best fitting cluster number was determined by comparing average silhouette coefficients for each possible cluster number. To find the OGT, we then plotted and analyzed the clusters visually.

### ﻿Fruiting

To investigate the conditions suitable for fruiting of the strains, we investigated both the formation of primordia, i.e. the very early developmental stage of mushrooms, and the formation of mature mushrooms. In this study we focused on fruiting body formation of basidiomycetes that form fruiting bodies directly from the mycelium, and thus the ascomycete *Morchellaimportuna* wild strains were not included in the fruiting experiments. For all basidiomycete strains, we first investigated primordial formation when grown on PDA and the basidiomycete strains sampled before 2024 (Table 1) were also investigated when grown on straw. In addition to this, we investigated primordia and fruiting body formation using customized protocols for individual strains.

The PDA approach was performed with the triplicates of each temperature from the mycelial growth phase. For the approach with straw, all basidiomycete strains were grown in duplicates at their OGT. We hydrated straw pellets (Svamphuset, Stockholm, Sweden) with 200 ml deionized water per 100 g pellets before sterilization. 9 g of the straw substrate were filled into petri dishes. The inoculation and cultivation conditions were the same as for the mycelial growth on PDA.

We chose the customized protocols with specific substrates (Table 2) based on the species’ ecology. Autoclaved substrates were filled into sterilized pipette tip boxes (13 × 9.5 × 6 cm, Sarstedt, Nümbrecht, Germany) and then sealed with micropore tape (Micropore, 3M, Saint Paul, USA). Per tip box, we used either 60.9 g sowing soil (Hasselfors Garden AB, Örebro, Sweden), 22.5 g alder chips (Millamore Premium, Harjumaa, Estonia), 43.5 g birch pellets (Svamphuset, Stockholm, Sweden) soaked with 2 ml deionized water per gram pellets, or 69 g rye flakes (Ekologiska Rågflingor, Axfood AB, Stockholm, Sweden). For this experiment, taking an exploratory approach, only one replicate was used. Inoculation and cultivation conditions were the same as in the other approaches.

All three approaches had the same change to fruiting conditions regarding temperature and light. The temperature was shifted down 5 °C after mycelial growth to induce fruiting initiation (Stamets 2000; Ford et al. 2015; Sakamoto 2018). After growing the mycelia in the dark, they were exposed to light at either 500 or 1’500 lux for fruiting (Perkins 1969; Ford et al. 2015; Sakamoto 2018). We chose a 12 h day and 12 h night regime and white light (Kamada et al. 2010; Wang et al. 2020). The cultures were switched to fruiting conditions when the mycelium was 0.5–1 mm away from the edge (Kües et al. 2016). The last condition switched for the fruiting phase, which only applied to the PDA and straw plates, was to remove the Parafilm® sealing to increase gas exchange (Kinugawa et al. 1994; Sakamoto 2018).

After switching plates or boxes to fruiting conditions, we observed on a daily basis whether primordia formation was present or not. As soon as primordia were visible, we documented the number of days since switching from mycelial growth to fruiting conditions. In case the primordia developed into mushrooms, we harvested them, dried them for 24 h at 70 °C, and weighed the total dry mass. This resulted in the three measures: 1) primordia presence, 2) fruiting time, and 3) mushroom dry mass. As not enough replicates were tested for each strain and condition, we could not perform a relevant statistical analysis.

**Table 2.** Temperature and substrates used in the customized part of the fruiting phase for each of the tested strains.

Strain	Temperature	Substrates
*Agaricusarvensis* MW41	20 °C	Sowing soil
*Armillariaostoyae* MW82	25 °C	Alder wood chips, birch pellets
*Coprinopsiscinerea* AmutBmut	30 °C	Sowing soil
*Coprinuscomatus* MW21	25 °C	Sowing soil
*Flammulinavelutipes* S311	25 °C	Alder wood chips, birch pellets
*Hericiumcoralloides* MW80	25 °C	Rye flakes, alder wood chips, birch pellets
*Hypholomalateritium* MW49	25 °C	Alder wood chips, birch pellets
*Lycoperdonperlatum* MW73	25 °C	Birch pellets
*Macrolepiotaprocera* MW90	25 °C	Sowing soil
*Marasmiusoreades* MW66	20 °C	Sowing soil, rye flakes
*Pleurotusostreatus* DkN001	30 °C	Alder wood chips
*Pleurotuspulmonarius* MW44	25 °C	Rye flakes, alder wood chips, birch pellets
*Strophariaaeruginosa* MW56	20 °C	Rye flakes, alder wood chips, birch pellets

### ﻿Strain handling and submission to culture collection

To maintain the strains, we used the established glycerol cryopreservation method (Linde et al. 2018). We added sterile glycerol (MP Biomedicals, Santa Ana, USA) to sterile ddH2O up to a 15% (v/v) solution. Mycelial cultures on PDA were cut to 3 × 3 mm cubes and five cubes were added to each tube. The cryotubes were then stored at -80 °C. Our newly isolated wild strains were also submitted to the Westerdijk Fungal Biodiversity Institute (CBS) culture collection (Table 1).

## ﻿Results

### ﻿Isolation of 17 edible saprotrophic wild strains

**Figure 2.**
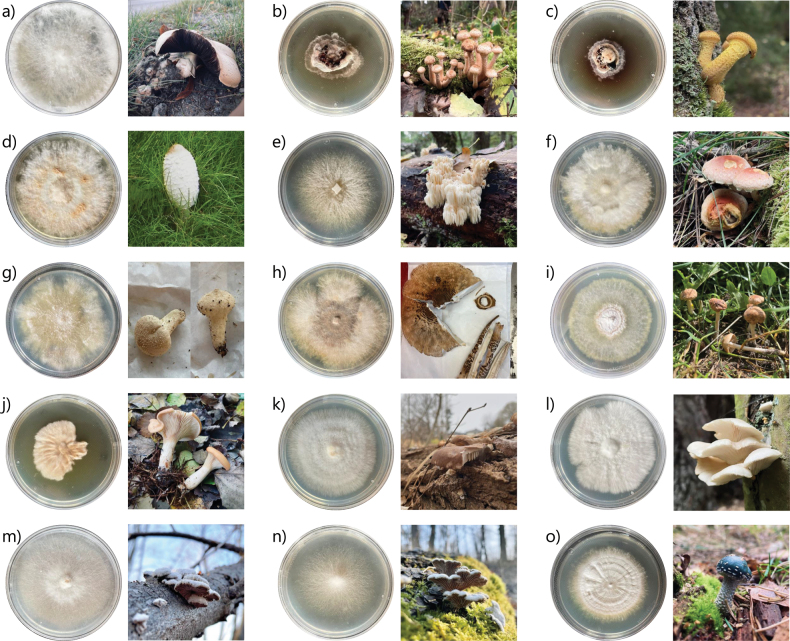
Mycelia on PDA (left) and mushrooms (right) of the 15 basidiomycete wild strains **a***Agaricusarvensis* MW41 **b***Armillariaborealis* MW40 **c***Armillariaborealis* MW82 **d***Coprinuscomatus* MW21 **e***Hericiumcoralloides* MW80 **f***Hypholomalateritium* MW49 **g***Lycoperdonperlatum* MW73 **h***Macrolepiotaprocera* MW90 **i***Marasmiusoreades* MW66 **j***Paralepistaflaccida* MW6 **k***Pleurotusostreatus* MW97 **l***Pleurotuspulmonarius* MW44 **m***Schizophyllumcommune* KK98 **n***Schizophyllumcommune* MW99 **o***Strophariaaeruginosa* MW56. Photos taken by Mario Walthert.

Of 33 mushrooms initially used for isolation, 17 resulted in isolated monohyphal cultures. Contamination by bacteria and different mold-forming fungi caused the exclusion of some isolation attempts. Molds were detected by the dust-like appearance of the often colored asexual spores (conidia) that are formed, and by a much faster growth on PDA compared to the target mushroom-forming species. From the ITS sequence analysis, all 17 wild strains were identified with high confidence (i.e., over 99% identity of the ITS sequence with a single species reported in GenBank) (Table 1), and the preliminary morphological species determination was confirmed in all cases. The distinct mycelium and mushroom characteristics of the 17 newly isolated wild strains are shown in Fig. 2.

### ﻿Strain - dependent variation in mycelia l growth rate

**Figure 3.**
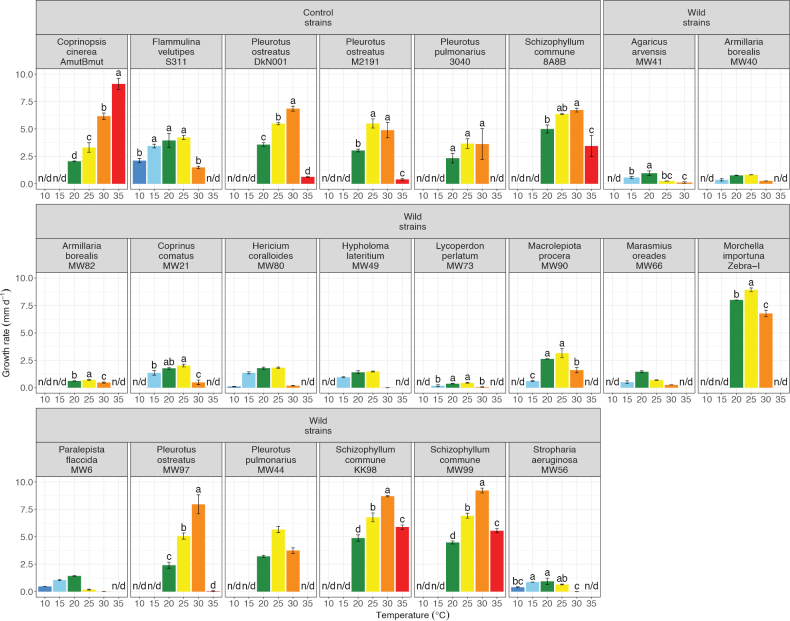
Average radial mycelial growth rates of all strains at tested temperatures. The strains were tested at a subset of 10, 15, 20, 25, 30, and 35 °C to find the optimal growth temperature (OGT). n/d stands for no data, i.e. temperatures that were not measured. Bars represent the average growth rate, while error bars represent the standard deviation from three replicate plates. Grouping letters based on Tukey multiple comparison of means (*CL* = 95%) are shown for the normal distributed strains.

**Figure 4.**
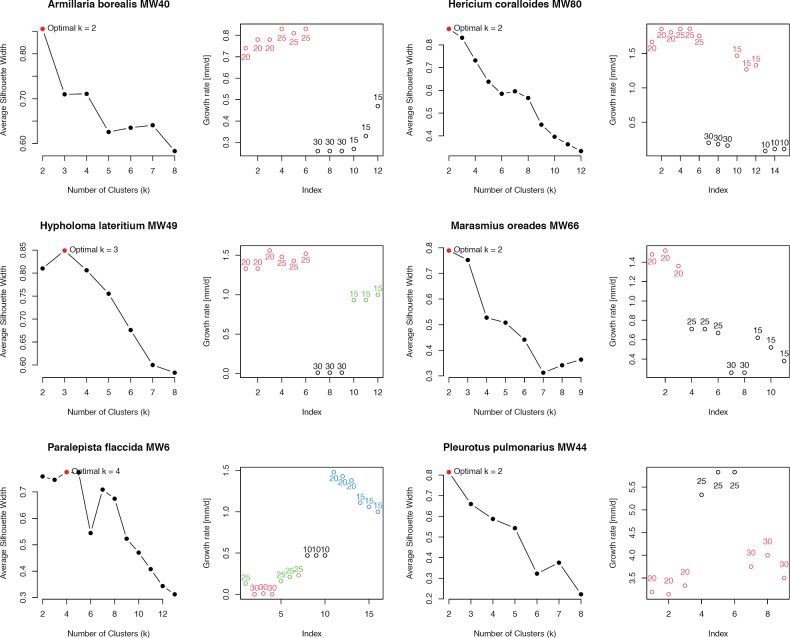
Silhouette cluster analysis for growth rates of all strains with non-normal distributed data. For each strain, indicated with species name and strain number, there are two graphs. The optimal cluster number analysis on the left shows the average silhouette width as a measure of fit for each tested number of clusters (k). The cluster graph on the right shows all data points and to which cluster they belong. Different clusters are shown by colors.

To find their optimal growth temperatures (OGT), the radial mycelial growth rate was measured at 20, 25, and 30 °C, and for most strains also at higher or lower temperatures (Fig. 3). Significant letter grouping from the Tukey test revealed the OGT for 15 strains. The remaining seven strains showed non-normal distributed growth rate data (Suppl. material 2: table S2). Therefore, their OGTs were determined with the cluster analysis (Fig. 4). Overall, we found a considerable variation between the growth rate of the newly isolated wild strains when grown at different temperatures (Fig. 3 and Suppl. material 2: table S3). The growth rates ranged from 0 mm d^-1^ (*SD* = 0) for *S.aeruginosa* MW56 at 30 °C up to 9.22 mm d^-1^ (*SD* = 0.22) for *S.commune* MW99 at 30 °C. In tendency, the control strains showed higher growth rates than the wild strains, particularly at 30 °C. However, the four wild strains from species also included as control strains and the only ascomycete, *M.importuna*, also showed fast growth and preference for warmer temperatures. Nevertheless, most wild strains grew better at 25 °C or even 20 °C (Fig. 3; Suppl. material 2: table S3).

Many strains grew faster at increasing temperatures up to certain optimal temperature, before the growth rate dropped sharply (Fig. 3; Suppl. material 2: table S3). For example, this was observed very clearly in *H.coralloides* MW80 and *P.flaccida* MW6. Focusing on the OGT, 9 out of 16 tested wild strains had their OGT at 25 °C (Figs 3, 4). Four wild strains grew better at 20 °C, and the three wild strains sampled in spring 2024 had their OGT at 30 °C. Several strains did show optimal growth over a range of multiple temperatures (Figs 3, 4). Most broad from the wild strains were *H.coralloides* MW80 and *S.aeruginosa* MW56 with an OGT range from 15 °C to 25 °C.

### ﻿Three wild strains produced primordia on PDA

**Figure 5.**
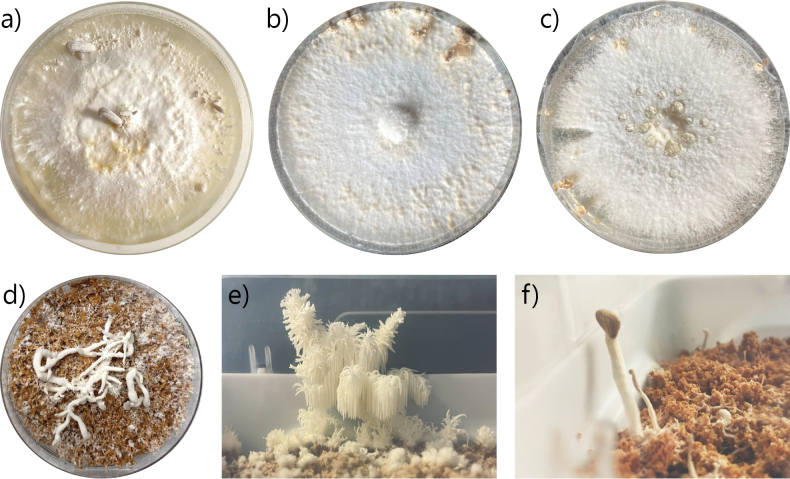
Primordia and mushroom development of wild strains. PDA plates: **a***Pleurotuspulmonarius* MW44 **b***Schizophyllumcommune* KK98 **c***Schizophyllumcommune* MW99. Plates with straw substrate: **d***Pleurotuspulmonarius* MW44. Boxes with birch substrate: **e***Hericiumcoralloides* MW80 **f***Pleurotuspulmonarius* MW44.

As mentioned before, the fruiting phase of this study was not statistically analyzed because not all conditions and strains were tested with a substantial replicate number. Nevertheless, the three measures of primordia presence, fruiting initiation time, and mushroom dry mass indicated trends for different strains. The control strains indicated that the fruiting conditions in this project enabled primordia and mushroom development (Table 3; Suppl. material 1: fig. S2). In tendency, faster-growing control and wild strains also developed primordia more often. From the wild strains, the fast-growers *P.pulmonarius* MW44 (Fig. 5a), *S.commune* KK98 (Fig. 5b), and *S.commune* MW99 (Fig. 5c) developed fruiting bodies on PDA. Additionally, *A.arvensis* MW41, *L.perlatum* MW73, and *M.oreades* MW66 did show the growth of some hyphal aggregates. However, it was not clear whether these structures were primordia, so these plates were scored as unsuccessful fruiting. None of the other wild strains showed any development at any of the tested conditions (Suppl. material 2: table S4).

Looking at the strains forming fruiting bodies on PDA, we did not observe a clear tendency for temperature and light conditions for primordia presence, fruiting initiation time, or mushroom dry mass. The formation rather depended on a strain-level, for example, *P.pulmonarius* MW44 indicated that it rather fruits at 1’500 lux than 500 lux (Table 3). The strains producing primordia needed between 8 and 41 days to initiate them. The two *S.commune* wild strains KK98 and MW99 were the fastest, particularly at the lowest tested temperature of 15 °C. Furthermore, *P.pulmonarius* MW44 was also rather fast in comparison with the control strains. *P.pulmonarius* MW44 was the only wild strain where the primordia developed into mushrooms, here defined as primordia that grew larger than 10 mm (Miles and Chang 2004). Its mushroom dry mass was in tendency higher at lower temperatures (Table 3).

**Table 3.** The fruiting results from the successfully fruited strains on potato dextrose agar (PDA).

Strain		Fruiting success^a^																	
		15 °C						20 °C						25 °C					
		500 lux			1’500 lux			500 lux			1’500 lux			500 lux			1’500 lux		
		Primordia^b^	Time^c^	Dry mass^d^	Primordia^b^	Time^c^	Dry mass^d^	Primordia^b^	Time^c^	Dry mass^d^	Primordia^b^	Time^c^	Dry mass^d^	Primordia^b^	Time^c^	Dry mass^d^	Primordia^b^	Time^c^	Dry mass^d^
Control strains	*Flammulinavelutipes* S311	-	-	-	2/3	30	79	-	-	-	3/3	32	50	0/2	n.p.	n.p.	0/1	n.p.	n.p.
	*Pleurotusostreatus* M2191	-	-	-	3/3	28	4	-	-	-	3/3	40	0	-	-	-	0/3	n.p.	n.p.
	*Pleurotusostreatus* DkN001	-	-	-	3/3	28	0	-	-	-	3/3	41	0	-	-	-	3/3	18	0
	*Pleurotuspulmonarius* 3040	-	-	-	3/3	23	0	-	-	-	2/3	22	0	2/2	26	0	1/1	14	0
Wild strains	*Pleurotuspulmonarius* MW44	-	-	-	3/3	25	16	-	-	-	3/3	23	8	1/3	11	2	3/3	31	0
	*Schizophyllumcommune* KK98	-	-	-	2/2	9	0	-	-	-	2/2	13	0	0/1	n.p.	n.p.	2/2	16	0
	*Schizophyllumcommune* MW99	-	-	-	1/2	8	0	0/1	n.p.	n.p.	1/2	13	0	0/1	n.p.	n.p.	0/2	n.p.	n.p.

^a^ A dash indicates that the condition was not tested, n.p. stands for no primordia on any plate for this condition. ^b^ Primordia presence is given as number of plates with successful fruiting body formation per total number of plates. ^c^ Time for initiation is given as average number of days from switch to fruiting conditions to first primordium. ^d^ The mushroom dry mass is given as average weight (mg) per plate.

### ﻿Straw led to more mushrooms

Similar to the assay from growth on PDA, the control strains showed that the chosen fruiting conditions were also enabling fruiting body formation on straw. Especially *Coprinopsiscinerea* AmutBmut underwent its full life cycle from inoculation to mature mushrooms within a week (Suppl. material 1: fig. S2). From the wild strains, *P.pulmonarius* MW44 produced coral-like mushrooms (Fig. 5d). All the other tested strains, that is all basidiomycetes, did not form fruiting bodies on straw. Some strains such as *L.perlatum* MW73 did not fully colonize the straw substrate and were thus not even switched to fruiting conditions.

### ﻿Specific subtrates resulted in additional fruiting success

The customized approach to fruiting consisted of testing strains of interest on sowing soil, rye flakes, alder chips, and birch pellets (Table 2). All strains tested on sowing soil colonized the substrate fully, but none developed fruiting bodies. No success resulted from rye flakes and alder wood chips. The mycelial cubes dried out within 48 h on both of these substrates and no growth was observed. The last substrate, birch pellets, was the most fruitful. All tested strains grew into the substrate, however to a varying extent. Full colonization of the birch substrate was reached by *P.pulmonarius* MW44 within three weeks, and by *H.lateritium* MW49 and *H.coralloides* MW80 within five weeks. From those strains, only *H.lateritium* MW49 did not develop fruiting bodies. *P.pulmonarius* MW44 produced primordia after two weeks, which developed into mushrooms (Fig. 5f). *P.pulmonarius* MW44 mushrooms developed only a small cap. Furthermore, fruiting body development in the *H.coralloides* strain MW80 was observed for the first time in this study. Its primordia grew after one week close to the tip box edge. They formed spiky structures after three more weeks and developed into the iconic and spectacular mushroom form (Fig. 5e) after another four weeks.

## ﻿Discussion

In this study, we isolated and characterized 17 Swedish strains from 14 saprotrophic fungal species that form edible mushrooms. These strains are made publicly available at CBS, and by providing this diverse new resource from natural environments to the community of mycologists, we open up for both further scientific studies and industrial development of mushroom-forming fungi. We chose to focus on edible fungi as these are particularly relevant for the food industry. Additionally, the newly isolated strains will contribute to the conservation of fungal genetic diversity and may also be of value for other industrial applications.

Our attempt to isolate mycelia from the mushrooms and to characterize their growth rates was in general successful. The tissue isolation method led to better results and was less laborious to obtain pure dikaryotic cultures compared to the spore print isolation method. However, the latter can be a useful backup method and should not be neglected. Contamination during the isolation phase could potentially be limited in the future by the use of a broader spectrum of antibiotics and the use of, e.g., benomyl to control growth of ascomycete molds (Bollen and Fuchs 1970). As a measure for fungal growth, we chose the radial growth rate. While this gives a measure for hyphal elongation and substrate colonization, it does not account for parameters such as colony density or hyphal width (Taniwaki et al. 2006). Nevertheless, the radial growth rate is a reliable and widely used proxy for fungal growth (Brancato and Golding 1953; Boddy 1983; Furlan 1997; Taniwaki et al. 2006; Krupodovora et al. 2021) and as the cultures grew uniformly and reproducibly, we consider the values of growth rate obtained in this study both relevant and comparative between the strains and species. By this approach, we found that the radial growth rate and OGT vary on a strain-level. Strains that belong to an established cultivation species tended to grow faster at their OGT compared to strains from species that are not commercially cultivated. This indicates that those species are widely used because their mycelium grows fast, thereby being able to rapidly colonize their substrates and initiate fruiting body development.

One of the most relevant contributions of our study is that we provide both novel strains and species that can be developed for mushroom production. For example, with *Morchellaimportuna* and *Paralepistaflaccida*, we contribute edible species new to accessible culture collections like CBS or American Type Culture Collection (ATCC) (see the Global Catalogue of Microorganisms; Wu 2013). Additionally, strains such as *Hericiumcoralloides* MW80 can contribute to the development of new species for commercial production of edible, saprotrophic fungal species that, to our understanding, are relatively rarely cultivated today. Our set of wild strains also comprises fast-growing strains of established species such as *Pleurotusostreatus*, *Pleurotuspulmonarius*, and *Schizophyllumcommune*. Generally, mushrooms can be a part of a healthy diet and contribute to providing the growing population with locally produced high-quality food (Miles and Chang 2004; Hyde et al. 2019; Sganzerla et al. 2022) and going beyond the few species that dominate the mushroom market is an attractive prospect for the development of a sustainable food production. However, it is worth mentioning that some of the species we describe here are commonly eaten in some regions of the world and not in others (Stamets 2000; Boa 2004; Miles and Chang 2004; Li et al. 2021), and hence, the food culture has to be open for new species to be successfully established on the market.

Mushrooms not only provide a healthy diet, but with their little water and space requirements they can be produced in an environment-friendly method and contribute to a circular economy (Grimm and Wösten 2018; Okuda 2022; Kržišnik and Gonçalves 2023). ﻿Even if growth and cultivation of mycorrhizal fungi like *Tuber* spp. can be successful (Coleman ﻿et al. 2024), it is less laborious to grow and cultivate non-symbiontic fungi (Beetz and ﻿Kustudia 2004; Chakravarty 2011; Sakamoto 2018). However, it is important to accept that the cultivation of different saprotrophic mushroom-forming fungi also can vary greatly in difficulty and success potential. For example, morel cultivation has gained much attention but still new strains need to be isolated, and additional knowledge on pest management and cultivation techniques is needed for an efficient production (Xu et al. 2022). Being a heterothallic ascomycete, it needs a fertilization step for fruiting, and our wild strains of *Morchellaimportuna* were therefore not included in our fruiting experiments (Liu et al. 2018; Du and Yang 2021), and for those we attempted to fruit, we were not able to bring all to form mushrooms. However, we identified basic growth and cultivation knowledge for the newly isolated wild basidiomycete strains. *Pleurotuspulmonarius* MW44 and the two *Schizophyllumcommune* strains KK98 and MW99 showed interesting growth characteristics for further application, in that they grew fast and developed fruiting bodies under multiple conditions. Together with *Pleurotusostreatus* MW97 they even outperformed, in terms of mycelial growth rate, their conspecific laboratory strains that are currently used in research and/or industry. Additionally, the strain *Hericiumcoralloides* MW80 demonstrated its potential and developed spectacular mushrooms. Other strains, for example, *Coprinuscomatus* MW21, *Macrolepiotaprocera* MW90, or *Marasmiusoreades* MW66, need more investigation regarding their cultivation conditions to optimize their growth and make them relevant for further development in mushroom production.

One of the most important cultivation conditions is the substrate. The agar medium we used, PDA, was shown to be suited for isolating wild strains from nature (Thawthong et al. 2014; Hoa et al. 2017). In the fruiting phase, however, fruiting body growth on PDA was often prematurely terminated, and the resulting mushroom deformed. PDA was overall a good baseline for the fruiting experiments, but straw and the customized approach were closer to conditions used for mushroom production (Beetz and Kustudia 2004; Miles and Chang 2004; Sánchez 2010; Grimm and Wösten 2018). Straw led to more mushrooms than PDA, but the coral-like structures for *P.pulmonarius* MW44 indicated that the conditions we used in this approach were suboptimal. Although light scarcity also comes into question, we hypothesize that the formation of coral-like mushrooms is related to excess CO_2_ (Kinugawa et al. 1994; Sánchez 2010). Aeration should be carefully controlled for in future attempts to enable successful fruiting.

From the four substrates used in the customized approach, the rye flakes and alder wood chips were too dry to enable colonization. They would need to be hydrated, and the use of rye grains instead of flakes, as well as alder sawdust instead of wood chips, would be closer to established conditions (Stamets 2000; Miles and Chang 2004; Grimm and Wösten 2018). For the soil substrate, the sowing soil was the available choice, but a more specific composition could help to fruit further strains. The birch pellets led to more success in cultivating mushrooms in this study. In particular, *H.coralloides* MW80 produced its characteristic and spectacular mushrooms. This success could be attempted to upscale in the food industry.

The results from the fruiting phase indicated that a more customized approach to the cultivation of new strains has the highest chance of succeeding. In case production were commercialized, we expect that a grain spawn run would help to make the process more effective. Grains such as rye would be inoculated with the pure culture and once colonized, used as an inoculate for the actual fruiting substrate (Stamets 2000; Miles and Chang 2004). This substrate composition can be inspired by the ecology of a species. However, a certain creativity is never wrong in the field of mushroom cultivation. Substrate recipes with mixtures of different components can often lead to the best fruiting results (Stamets 2000; Miles and Chang 2004; Hoa et al. 2017). Systematic testing is the most suited approach in cultivation of fungi, where scientific knowledge is scarce.

The value of our diverse sample made available in this study can go beyond development of new species for mushroom production. Specifically, wild strains also bear a diversity in mycelial growth characteristics and/or morphology, leading to a broader application potential (Adrio and Demain 2003; Kržišnik and Gonçalves 2023). For example, ﻿fungi growing fast at low temperatures could be used in mycelium-based technologies with ﻿less heating energy consumption. We show such distinct growth characteristics in this study ﻿for the wild strains isolated from Swedish nature, in that, for example, some of the local Swedish strains (*Hericiumcoralloides* MW80, *Paralepistaflaccida* MW6, and *Strophariaaeruginosa* MW56) ranged in their optimal growth to temperatures as low as 15 °C, potentially indicating an adaptation to the Nordic climate. Furthermore, the rising interests in fungal-based food and local products (Hellwig 2023) can be combined when a diverse set of fungal strains sampled from different parts of the world are used in the food industry.

Finally, apart from industrial and research approaches, culture collections are also crucial for fungal conservation (Sette et al. 2013). We are facing a biodiversity crisis, that requires actions, and this embraces also fungi (Mueller et al. 2022; Antonelli et al. 2023). Although fungi have many crucial roles in our ecosystems and comprise approximately 20% of all eukaryotic species, they are understudied, and their conservation does not receive enough attention (Niskanen et al. 2023). Non-invasive sampling, isolating, and storing strains from nature, as done in this study, is one of the most effective ways to protect fungal species by making them available for re-establishment in natural environments (Niskanen et al. 2023). Additionally, this procedure is following the access and benefit-sharing provisions of the Convention on Biological Diversity (CBD), designed to ensure that the physical access to genetic resources is facilitated and that the benefits obtained from their use are shared equitably with the providers.

## ﻿Conclusion

Both wild and cultivated fungi can be resources of low-calorie functional food. Isolating new fungal strains from nature bears the potential of finding strains with desired growth characteristics that can help humans to produce enough high-quality food and to create a more circular economy. With this study we contribute to the collection of wild edible mushrooms collected from nature that enable further applications. Only five of the species in our study had Swedish strains available previously in widely available culture collections such as CBS and ATCC (Wu et al. 2013). Three species were even entirely new to CBS and thus contribute to its standing as one of the most comprehensive fungal genetic resource collections (Hawksworth 2004). Furthermore, some of these wild strains showed growth rates and fruiting properties that make them interesting for further use. Hence, this study offers a major contribution to the collections of natural strains of potentially economically important fungi.

Be it in the food industry, research, biotechnology, art, or society in general, interest in fungi is increasing. Fungi generate high hopes about being the source of sustainable solutions and accompany us on our way to a greener future. In the light of sustainability, the cultivation of local strains is of high importance to industry and society. Their isolation and cultivation represent a first step on the way to application.

## ﻿Additional information

### Author ORCIDs

Hanna Johannesson https://orcid.org/0000-0001-6359-9856

### Data availability

The datasets used and/or analyzed during the current study are available from the corresponding author on reasonable request.

## Supplementary materials

Supplementary material 1 Supplementary figures Data type pdf Explanation note **figure S1.** The two methods used in this study to obtain pure dikaryotic mycelial cultures of basidiomycetes. The tissue isolation method is shown on the left, the spore print isolation method on the right. The scheme is not drawn in size scale. **figure S2.** Primordia and mushroom development of control strains. PDA plates: **a***Flammulinavelutipes* S311 **b***Pleurotusostreatus* M2191 **c***Pleurotusostreatus* DkN001 **d***Pleurotuspulmonarius* 3040. Plates with straw substrate: **e***Coprinopsiscinerea* AmutBmut **f***Pleurotusostreatus* DkN001 **g***Pleurotuspulmonarius* 3040. Box with birch substrate: **h***Flammulinavelutipes* S311. https://binary.pensoft.net/file/1253398This dataset is made available under the Open Database License (http://opendatacommons.org/licenses/odbl/1.0/). The Open Database License (ODbL) is a license agreement intended to allow users to freely share, modify, and use this Dataset while maintaining this same freedom for others, provided that the original source and author(s) are credited.Mario Walthert, Markus Hiltunen Thorén, Hanna Johannesson

Supplementary material 2 Supplementary tables Data type xlsx Explanation note **table S1.** Information on the six control strains used in this project. **table S2.** Results of Shapiro-Wilk test and Silhouette cluster analysis for control and wild strains. **table S3.** Average growth rates of all strains for each tested temperature on PDA. **table S4.** The fruiting results from all strains on potato dextrose agar (PDA). https://binary.pensoft.net/file/1253399This dataset is made available under the Open Database License (http://opendatacommons.org/licenses/odbl/1.0/). The Open Database License (ODbL) is a license agreement intended to allow users to freely share, modify, and use this Dataset while maintaining this same freedom for others, provided that the original source and author(s) are credited.Mario Walthert, Markus Hiltunen Thorén, Hanna Johannesson
