# Supplementary material for: ﻿Isolation and characterization of edible mushroom-forming fungi from Swedish nature
Source: IMA Fungus. 2025 Feb 17;16:e142215. doi: 10.3897/imafungus.16.142215 (PMC11882028; doi:10.3897/imafungus.16.142215)
Supplement: Supplementary material 1 — Supplementary figures [file imafungus-16-e142215-s001.pdf]

Supplemental Figures

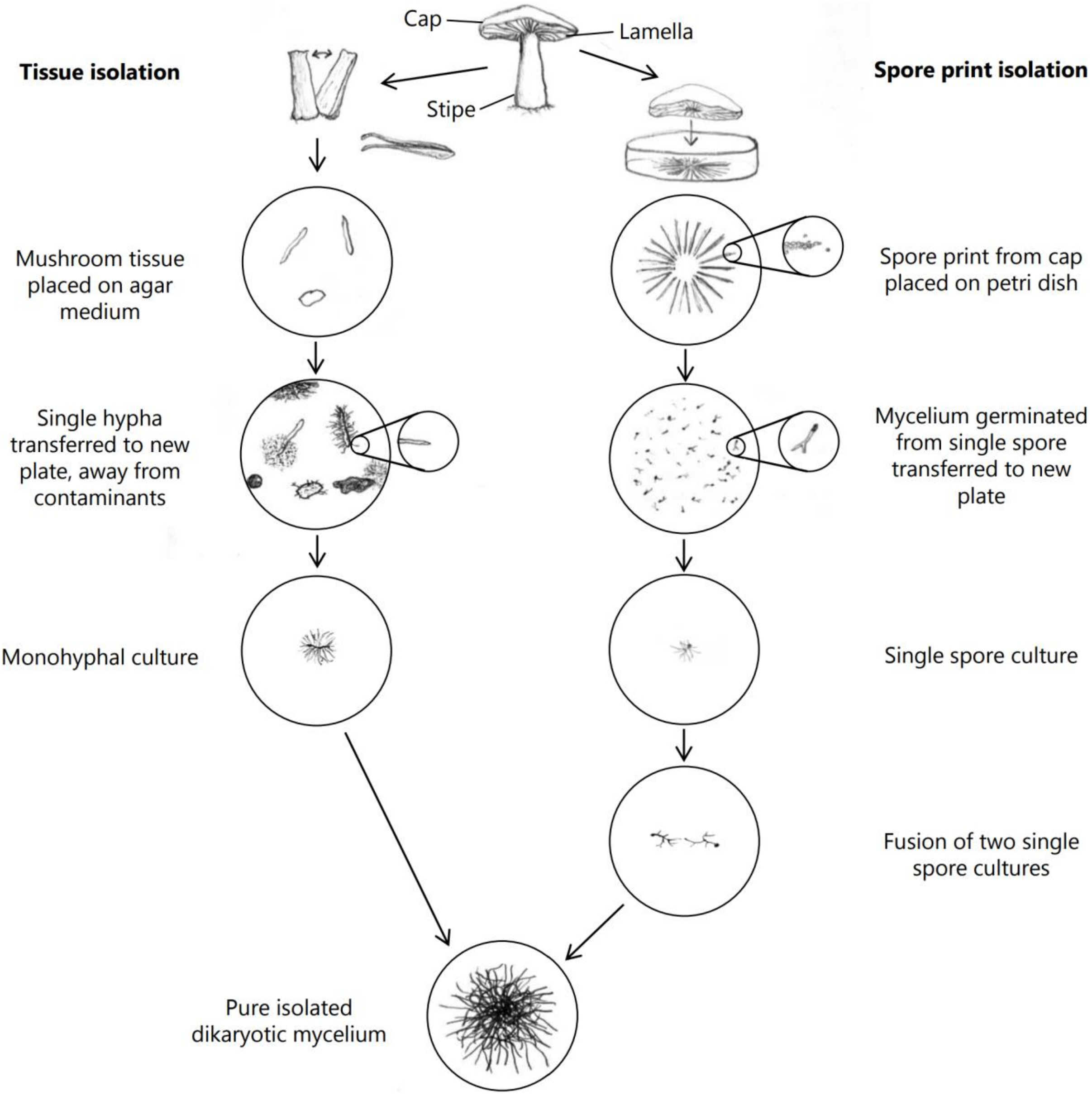

Supplemental Figure S1. The two methods used in this study to obtain pure dikaryotic mycelial cultures of basidiomycetes.

The tissue isolation method is shown on the left, the spore print isolation method on the right. The scheme is not drawn in size scale.

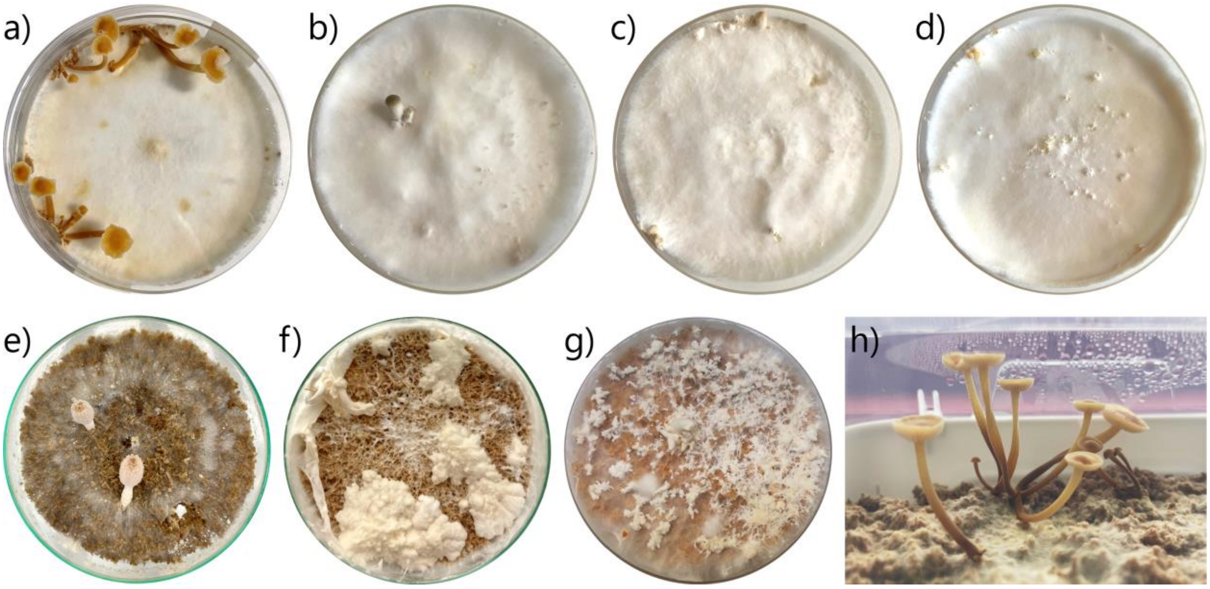

Supplemental Figure S2. Primordia and mushroom development of control strains.

PDA plates: a) *Flammulina velutipes* S311. b) *Pleurotus ostreatus* M2191. c) *Pleurotus ostreatus* DkN001. d) *Pleurotus pulmonarius* 3040. Plates with straw substrate: e) *Coprinopsis cinerea* AmutBmut. f) *Pleurotus ostreatus* DkN001. g) *Pleurotus pulmonarius* 3040. Box with birch substrate: h) *Flammulina velutipes* S311.
